# Systematic Review of Cancer Research Output From Africa, With Zambia as an Example

**DOI:** 10.1200/GO.21.00079

**Published:** 2021-06-02

**Authors:** Violet Kayamba, Wilbroad Mutale, Holly Cassell, Douglas Corbett Heimburger, Xiao-Ou Shu

**Affiliations:** ^1^ Tropical Gastroenterology and Nutrition Group, Department of Internal Medicine, Lusaka, Zambia; ^2^ University of Zambia School of Medicine, Lusaka, Zambia; ^3^ University of Zambia School of Public Health, Lusaka, Zambia; ^4^ Vanderbilt Institute for Global Health, Vanderbilt University Medical Center, Nashville, TN; ^5^ Division of Epidemiology, Department of Medicine, Vanderbilt Epidemiology Center, Vanderbilt-Ingram Cancer Center, Vanderbilt University Medical Center, Nashville, TN

## Abstract

Cancer occurrence is increasing in Africa, although research has lagged. The objective of this review was to analyze cancer research outputs from Africa, with a particular focus on Zambia.

**METHODS:**

We searched PubMed for published cancer-related articles from African countries. All articles reporting on cancer in Africa were considered. We conducted analyses to explore correlations between cancer research output and total population, gross domestic product, and new cancer cases recorded in 2020. For Zambia articles, we also analyzed cancer types and time trends.

**RESULTS:**

A total of 48,487 cancer-related publications from Africa were identified, with nearly half coming from Egypt (13,372; 28%) and South Africa (9,393; 19%). Cancer research output correlated significantly with country population (Spearman's correlation coefficient 0.74; *P* < .001) and the number of new cancer cases recorded in 2020 (Spearman's correlation coefficient 0.77; *P* < .001). Standardized by population size, Western Sahara (0.576), Seychelles (0.244), Tunisia (0.239), South Africa (0.158), and Egypt (0.131) had the highest overall output per 1,000 population. A total of 244 publications were from Zambia; the most studied cancers were cervical (25%), Kaposi sarcoma (24%), and breast (10%). Although an increase in cancer research output from Zambia was noted, only 33% of publications were first or last authored by Zambians. The major limitation of this review is that the evaluation was based on a single electronic database, PubMed.

**CONCLUSION:**

Cancer research output from Africa is very low, with many of the publications concentrated in a few countries. There is an urgent need to invest in both human resources and infrastructure to increase cancer research output from African countries, particularly in less populous countries.

## INTRODUCTION

Africa is experiencing an unprecedented increase in cancer cases,^1^ with incidence rates expected to double by 2040.^2^ With a population more than 1.3 billion, GLOBOCAN reported an estimated 1,109,209 new cancer cases and 711,429 cancer-related deaths in 2020 in Africa.^2,3^ The most common cancers in Africa are prostate, liver, colorectal, lung, and non-Hodgkin lymphoma in males and breast, cervical, colorectal, liver, and ovarian in females.^2^ Zambia is a landlocked, lower-middle–income African country located in the southern part of the continent with a population of about 18 million.^3^ Estimates for 2020 show that Zambia had at least 13,831 new cancer cases and 8,672 cancer deaths.^2^ Among Zambian males, the most common cancers are prostate, Kaposi sarcoma, esophageal, liver, and colorectal, whereas in females, cervical, breast, Kaposi sarcoma, colorectal, and non-Hodgkin lymphoma predominate.

**CONTEXT**

**Key Objective**
The main objective of this review was to analyze cancer-related publications from Africa that are available on the electronic database PubMed with particular focus on Zambia.
**Knowledge Generated**
Cancer research output from Africa is very low and currently dominated by South Africa and Egypt. Overall, the number of published articles from African countries correlates with population size but not the current gross domestic product.
**Relevance**
With such low research output, there are limited data on the occurrence and characteristics of cancer in Africa. There is need to employ deliberate efforts aiming at encouraging and facilitating cancer-related publications from Africa.

Medical research is an avenue of information acquisition on the burden, causation, and prognosis of disease, and medical research output is one of the benchmarks of success. The output of cancer research from Africa has generally been low but is slowly gaining momentum.^4^

In Zambia, there have been some strides toward improvement of cancer research funding, most of which is provided by international funders. The 2017 countrywide report, for example, demonstrated a high prevalence of non-communicable disease risk factors and emphasized the need to prioritize prevention and control measures.^5^ These goals, however, cannot be attained without an understanding of the etiologic factors influencing disease development and underlying biologic mechanisms, which can only be provided by rigorous cancer research. To assess the current status of cancer research output in Zambia, we conducted a comprehensive assessment of literature from Zambia, on the basis of the electronic database PubMed. We further evaluated the time trends of cancer research output.

## METHODS

We searched the PubMed electronic database on December 15, 2020, using search queries for cancer types and African country names as indicated in the Data Supplement. The keywords were used individually or in combination to search for cancer-related publications in English. We included in the analysis all published articles reporting on cancer. When searching for outputs from African countries, we did not evaluate actual content in detail and therefore did not use any exclusion criteria.

We then interrogated the database by searching for publications from Zambia, using the same cancer-related search terms (Data Supplement). Identified article titles were individually reviewed for relevance. Those not directly related to cancer were excluded. We then reviewed available abstracts of selected publications to determine study types and confirm that articles were reporting Zambian data. We further evaluated authors of all selected articles. Using our knowledge of Zambian names and individuals involved in cancer research throughout the country, we identified Zambian authors listed in the publications. Searching and evaluation of articles were done by the first author.

Information on gross domestic product (GDP) per capita in US dollars was obtained from the International Monetary Fund data for 2019.^6^ In addition, we used the World Bank website to gather population data for individual African countries.^3^ Data on new cancer cases recorded in 2020 were obtained from the GLOBOCAN website.^2^

This review was done in accordance with Preferred Reporting Items for Systematic Reviews and Meta-Analyses guidelines.

### Statistical Analysis

Data were analyzed using STATA version 15 (College Station, TX). We used proportions and percentages to summarize the data. Continuous variables such as number of publications, population size, or GDP were checked for normal distribution using the Shapiro-Wilk test. Spearman's rank correlation coefficient (ρ) was applied to estimate correlation coefficients between the number of publications with GPD and size of population. For categorical variables, we employed the Kruskal-Wallis test. In all cases, a two-sided *P* value < .05 was considered statistically significant.

## RESULTS

### Cancer Research Output From Africa

**FIG 1:**
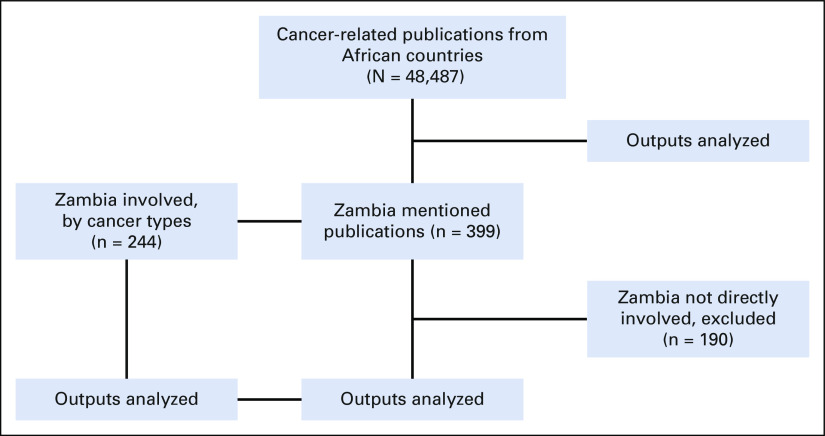
Flowchart illustrating the selection of publications from the PubMed database for the analysis.

**FIG 2:**
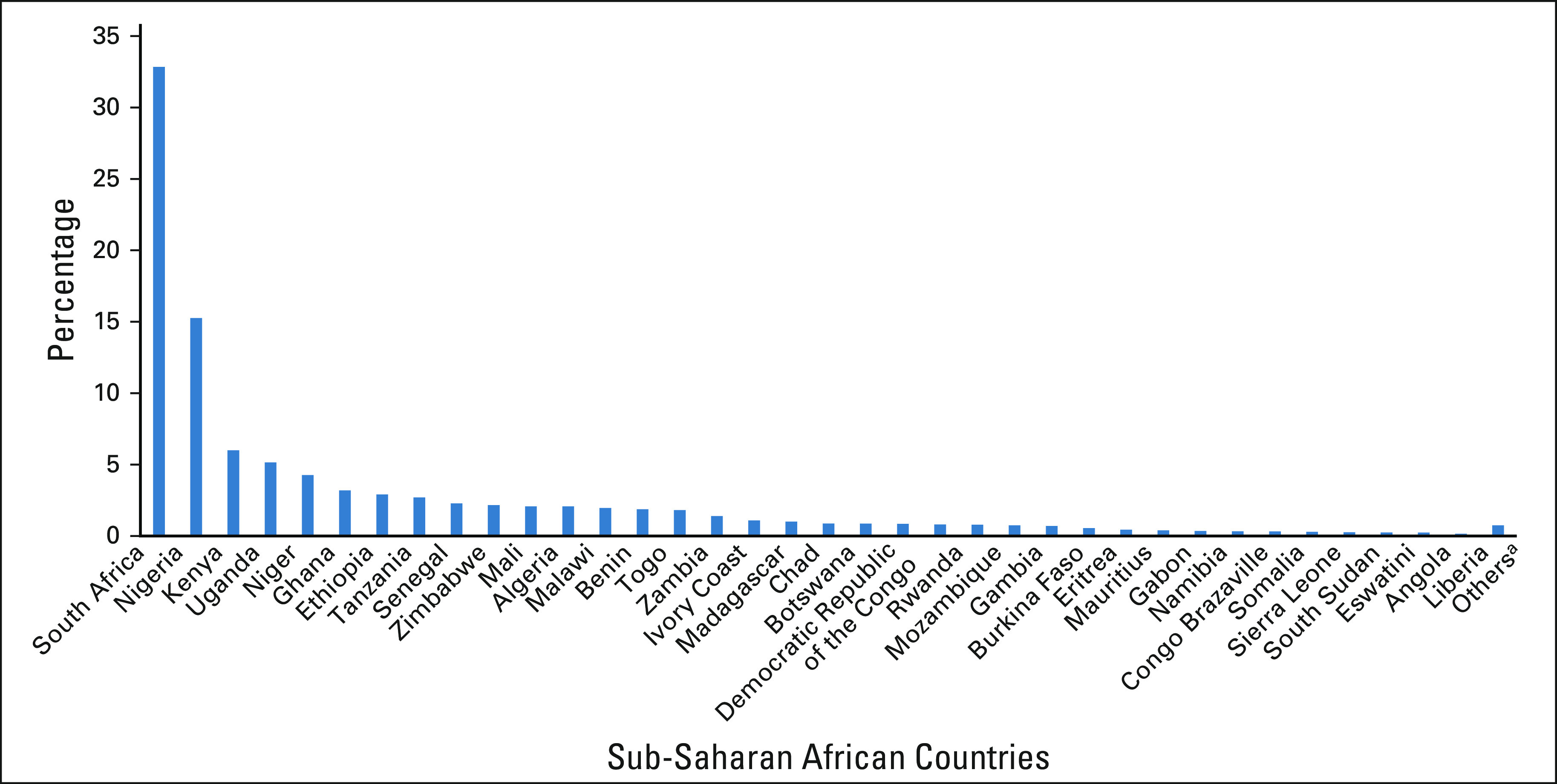
Percentage of cancer research publications from sub-Saharan Africa on the basis of the electronic database PubMed. ^a^Included among others are Mauritania, Guinea-Bissau, Lesotho, Burundi, Seychelles, Cape Verde, Djibouti, Comoros, and Sao Tome and Principe.

The search for cancer-related publications from 52 African countries yielded 48,487 articles (Fig 1). The earliest publication from Africa was in 1947. Egypt had the highest number of publications at 13,372 (28%), followed by South Africa at 9,393 (19%) (Table 1). Fourteen countries had < 100 cancer-related articles on PubMed. Zambia was at position 16 within sub-Saharan Africa, listing 399 publications, whereas Djibouti had only 15 publications (Fig 2). We could not analyze publications from the Central African Republic or Equatorial Guinea, as the multiple words in these country names made it difficult to select publications that were exclusively from these countries.

**TABLE 1:**
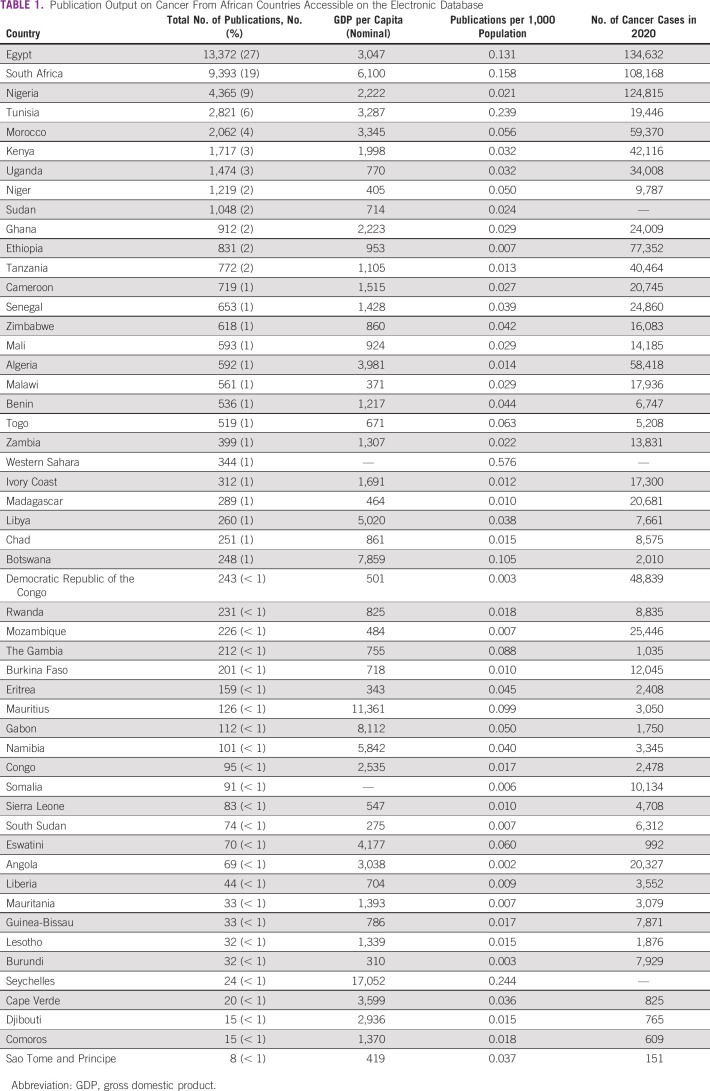
Publication Output on Cancer From African Countries Accessible on the Electronic Database

Standardized by population size, the countries with the highest cancer-related publications per 1,000 population were Western Sahara (0.576), Seychelles (0.244), Tunisia (0.239), South Africa (0.158), and Egypt (0.131). Countries with the lowest publications per 1,000 population were Angola (0.002), Burundi (0.002), Democratic Republic of Congo (0.003), and Somalia (0.006) (Table 1). Zambia was in position number 29 with 0.022 publications per 1,000 population. There was a significant positive correlation between country population and number of cancer-related publications (Spearman's ρ 0.74; *P* < .001). Similarly, there was a statistically significant correlation between cancer research output and the number of new cases in 2020 (Spearman's ρ 0.77; *P* < .001). However, cancer research output was not correlated with country's GDP (Spearman's ρ 0.02; *P* = .90). Some countries with very low GDP, such as Niger, had much higher cancer publication output than those with relatively higher GDP, such as Gabon, Seychelles, or Mauritius.

Categorizing these three variables (GDP, population, and cancer cases in 2020) into quartiles showed similar patterns, that is, cancer research output increased with increasing population and new cancer cases in 2020 (*P* < .001; Table 2). No significant association was observed between country's GDP and cancer research output (*P* = .54; Table 2).

**TABLE 2:**
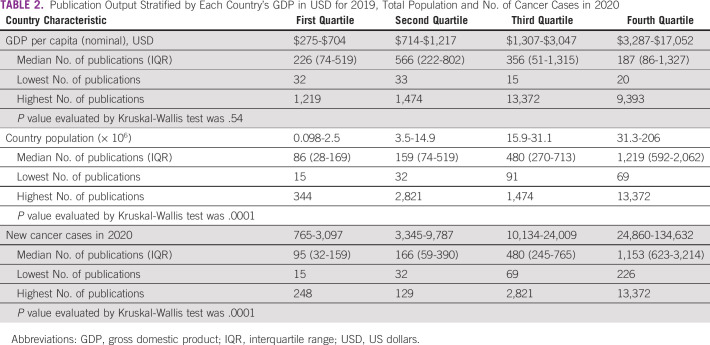
Publication Output Stratified by Each Country's GDP in USD for 2019, Total Population and No. of Cancer Cases in 2020

### Cancer Research Output From Zambia

**FIG 3:**
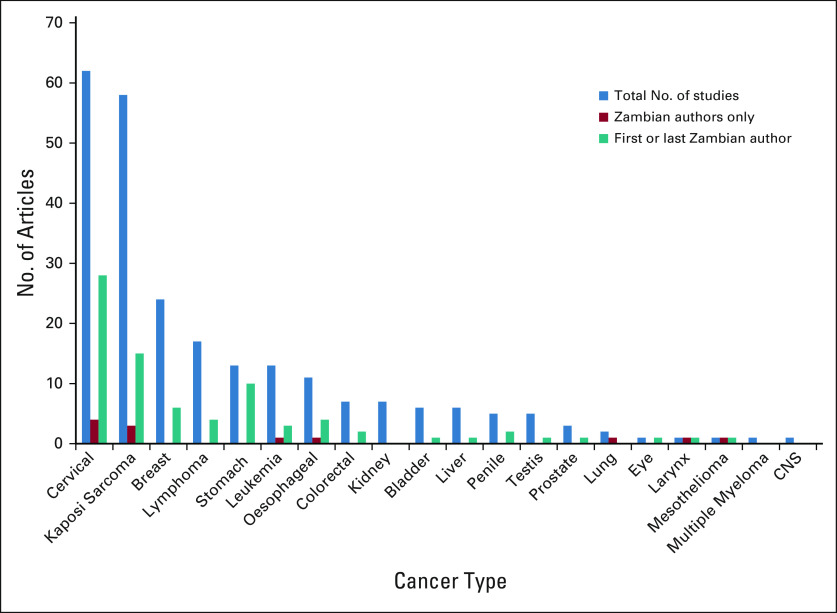
Cancer-related publications from Zambia by cancer type and by authorship.

We found 399 cancer-related publications from Zambia, with the first being from 1966. Among these, 244 publications were selected as directly reporting data from Zambia (Fig 1). Of these, 45 (18%) were reviews, 18 (7%) were clinical trials, 8 (3%) were case-control studies, 13 (5%) were cohort studies, and 70 (29%) were cross-sectional studies. There were 19 (8%) studies that focused on basic sciences and 32 (13%) case reports. The remaining 39 (16%) studies were either surveys or editorials or had study designs that were not clearly outlined. Analysis by cancer type showed that the highest number of publications was for cervical cancer (62; 25%), closely followed by Kaposi sarcoma (58; 24%) (Fig 3). Eighty-one (33%) publications from Zambia had either a first or last Zambian author. For publications on stomach cancer, 10 of 13 (77%) had a Zambian as the first author. Only 12 (5%) publications had exclusively Zambian authors (Fig 3).

### Time Trends for Cancer Research Output From Zambia

**FIG 4:**
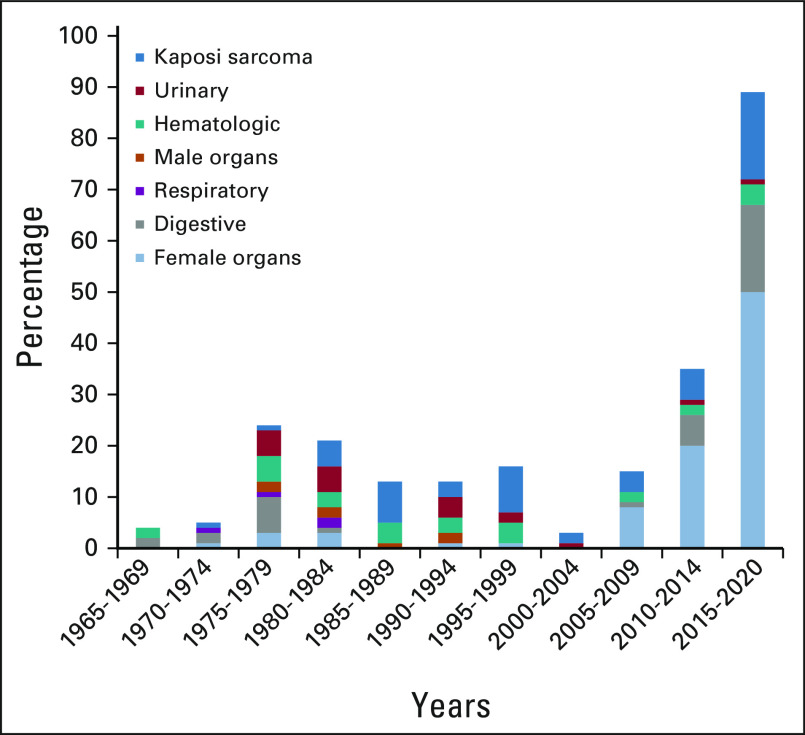
Time trends for cancer research output from Zambia by cancer sites.

There was a sharp increase in publications between 1975 and 1979, followed by a gradual decrease, which reached a nadir between 2000 and 2004. From 2005, research output once again increased and has been sustained since (Fig 4). Analysis by cancer type showed a dramatic increase in publications on cancers of the female reproductive organs, most of which focused on cervical cancer research. There were some publications on digestive cancers leading up to 1984 and then none until following the turn of the century. There were no publications on respiratory cancers since the early 1980s. Similarly, the last publications on male reproductive cancers were from the early 1990s (data not shown). Conversely, publications on Kaposi sarcoma have been relatively consistent throughout the years evaluated, with the highest numbers between 2015 and 2019 (Fig 4).

## DISCUSSION

We evaluated cancer research output from Africa, with a particular focus on Zambia. Using published articles from PubMed, we were able to review the total number of articles published and correlate them with country populations, GDP, and cancer cases reported in 2020. We found that Egypt and South Africa produced the highest number of cancer-related publications, whereas most remaining countries had very low research output. There was a strong positive correlation between publication output and population size, however. For Zambia, we found an overall increase in cancer-related research output, mainly after 2015. However, very few of these studies were led by Zambians.

Increased cancer research in Africa is key to responding to the current and predicted increase in cancer burden and the foundation for the development and successful implementation of evidence-based strategies for disease prevention and control.^7^ Efforts directed at increasing cancer research output, however, are hampered by several obstacles throughout the continent, such as a lack of infrastructure, trained personnel, research funding, and the ever-increasing demand for meager resources.^1^ Historically, cancer research training and funding have not been top priorities for many African governments. However, there are examples of countries that recently embarked on establishing national research funding schemes^8^ in a quest to improve output, but such efforts are still in their infancy, and most countries are still lagging. The intention to increase research funding has been expressed, with many African governments having non-communicable diseases on their national health agendas, but with very modest practical implementations of such plans.^9,10^

Factors compromising cancer research output from African countries are multifaceted, with the major one being a lack of resources. There is very little research funding from private enterprise in Africa, and most funding is from government or international funders.^11^ Nevertheless, there is hope for improving cancer research in Africa, with several national and international partners working toward this goal. The African Organisation for Research and Training in Cancer is one example. African Organisation for Research and Training in Cancer was established to improve cancer research in Africa, support oncology training programs, and deal with the challenges of creating cancer control programs with appropriate public awareness.^12^ In addition, there are several international funding bodies with calls directed at encouraging cancer research in Africa, particularly among early career individuals.^7^

Our results have demonstrated obvious research output disparities among African countries. Other researchers have shown evidence that countries with higher domestic expenditures in research and development, such as Egypt, Morocco, South Africa, and Tunisia, had much higher publication output.^11^ Evidence has also reported that the most populous African countries, such as Nigeria, South Africa, and Egypt, have higher research productivity.^13^ Our results showed a similar pattern. However, publication output standardized by population size showed a slightly different picture. Relatively higher per capita publication output was seen in less populous countries such as Western Sahara and Seychelles, so the linkage to countries' wealth is not straightforward. GDP, a commonly referenced measure of population well-being, has some limitations, with minimal consideration of sociocultural, ecologic, and collective discourses, which could be construed as part of general well-being.^14^ We found no clear link between cancer-related publications and GDP. It is possible that this observation could be partially explained by more international research funding to countries with lower GDP, but if true, this assertion requires more rigorous scrutiny. Our findings emphasize the need to consider all these factors when evaluating research output.

Africa has relatively few active researchers and continues to suffer from the problem of skilled workers emigrating to more developed centers.^11^ To curb the African brain drain, there are several funding mechanisms set up to encourage skilled African researchers to accept positions in institutions in their home countries and contribute to improving local capacity.^15^ Investing in African institutions for research training is another strategy that has shown efficacy in improving the retention of African graduates.^16,17^ The international ranking for many African universities is very low. The two countries with the highest cancer research output, Egypt and South Africa, are also the best represented in the Times Higher Education World University Rankings.^18^ Thus, establishing excellent research training programs and retaining and cultivating future mentors for research in major universities in Africa are top priorities to enhance cancer research and output. It is true that high-impact articles often require broad international collaborations, and many such publications were previously done with partners outside of Africa.^11^ This tradition should continue. However, such research collaborations are often driven by funding opportunities.

Research output is generally more visible when published in credible peer-reviewed journals. A few years ago, Kebede et al^19^ conducted a survey of health research institutions in 42 African countries and found that books or book chapters accounted for the highest number of informational products published from Africa. Therefore, measuring research output using peer-reviewed published articles may not be a true representation of African research output. There is, thus, a need to conform to international practices and make such information available in peer-reviewed journals. To encourage cancer publications from low-resource settings, there has been an emergence of journals focused on oncology in global health, such as journal ASCO's *JCO Global Oncology*, among others.^20,21^

Research dynamics for Zambia have shown an overall increase in output over the past decade. The most notable increase was in HIV-related cancers, resulting from an influx of research investments. Unfortunately, such success has not yet been extended to research on other cancers. For example, the third most researched cancer, breast cancer, leveraged resources that were initially set up for cervical cancer research, which, in turn, evolved from a well-established HIV program in Zambia.^22^ It remains unclear why research focusing on male organs, such as prostate research, was high in the 1970s and 1980s and completely disappeared thereafter. Similarly, there were virtually no published articles on digestive cancers between 1982 and 2008. We can only speculate that the focus at that time shifted to the then emerging HIV pandemic, which overwhelmed the sector, a dynamic that has since been altered by antiretroviral therapy. GI Kaposi sarcoma, for example, showed a steep rise in the 1980s and 1990s and steadily dropped following the implementation of antiretroviral therapy programs, beginning in 2005.^23^

Evaluating the positions of authorship from Zambian publications gave us an idea on the roles of indigenous Zambians in improving research output. Overall, only one third of publications had either first or last author positions held by Zambians, suggesting that they had taken on leadership roles in the research.

Our study had several limitations, which could compromise its validity. First, we searched only PubMed and, therefore, did not analyze peer-reviewed publications, which were not archived there. Second, some articles we examined focused on more than one cancer. Therefore, when evaluating outputs by cancer type from Zambia, these articles could have been counted more than once. Third, we did not evaluate the content of the studies in detail, so we could not comment on their quality. Fourth, the GDP values we used were the current values, so they did not reflect potentially varying GDPs during the period under review. Fifth, ascertainment of Zambian authors was merely based on our knowledge of Zambian names and not on objective information. Some authors could have been misclassified. Finally, we did not analyze research investments of African governments, the prevalence and nature of African research training programs, or the numbers of trained researchers, which may vary widely across the continent and would likely correlate with research output.

However, our review highlights the low cancer-related research output from Africa, a problem that has potential implications on cancer programs, as cancer across the continent evolves over time.

In conclusion, cancer research output from Africa is very low relative to the probable, with some highly populous countries contributing the majority of publications. Research results drawn from those countries may not necessarily be generalized to other African countries.^24^ The link between GDP and cancer research output is weak and needs further evaluation. There is a need to invest more resources in non–HIV-associated cancer research in Zambia.
